# Evaluative reports on medical malpractice policies in obstetrics: a rapid scoping review

**DOI:** 10.1186/s13643-017-0569-5

**Published:** 2017-09-06

**Authors:** Roberta Cardoso, Wasifa Zarin, Vera Nincic, Sarah Louise Barber, Ahmet Metin Gulmezoglu, Charlotte Wilson, Katherine Wilson, Heather McDonald, Meghan Kenny, Rachel Warren, Sharon E. Straus, Andrea C. Tricco

**Affiliations:** 1 Knowledge Translation Program, Li Ka Shing Knowledge Institute, St. Michael’s Hospital, 209 Victoria Street, East Building, Toronto, Ontario M5B 1W8 Canada; 2 World Health Organization Kobe Centre, Kobe, Japan; 3 Department of Reproductive Health and Research, World Health Organization, Genève, Switzerland; 4 Department of Geriatric Medicine, University of Toronto, Toronto, Ontario Canada; 5 Epidemiology Division, Dalla Lana School of Public Health, University of Toronto, Toronto, Ontario Canada

**Keywords:** Litigation, Obstetrics, Medical malpractice, Medical liability, Costs

## Abstract

**Background:**

The clinical specialty of obstetrics is under particular scrutiny with increasing litigation costs and unnecessary tests and procedures done in attempts to prevent litigation. We aimed to identify reports evaluating or comparing the effectiveness of medical liability reforms and quality improvement strategies in improving litigation-related outcomes in obstetrics.

**Methods:**

We conducted a rapid scoping review with a 6-week timeline. MEDLINE, EMBASE, LexisNexis Academic, the Legal Scholarship Network, Justis, LegalTrac, QuickLaw, and HeinOnline were searched for publications in English from 2004 until June 2015. The selection criteria for screening were established a priori and pilot-tested. We included reports comparing or evaluating the impact of obstetrics-related medical liability reforms and quality improvement strategies on cost containment and litigation settlement across all countries. All levels of screening were done by two reviewers independently, and discrepancies were resolved by a third reviewer. In addition, two reviewers independently extracted relevant data using a pre-tested form, and discrepancies were resolved by a third reviewer. The results were summarized descriptively.

**Results:**

The search resulted in 2729 citations, of which 14 reports met our eligibility criteria. Several initiatives for improving the medical malpractice litigation system were found, including no-fault approaches, patient safety policy initiatives, communication and resolution, caps on compensation and attorney fees, alternative payment system and liabilities, and limitations on litigation.

**Conclusions:**

Only a few litigation policies in obstetrics were evaluated or compared. Included documents showed that initiatives to reduce medical malpractice litigation could be associated with a decrease in adverse and malpractice events. However, due to heterogeneous settings (e.g., economic structure, healthcare system) and variation in the outcomes reported, the advantages and disadvantages of initiatives may vary.

**Electronic supplementary material:**

The online version of this article (10.1186/s13643-017-0569-5) contains supplementary material, which is available to authorized users.

## Background

The current medical malpractice system is costly and inefficient [1]. The clinical specialty of obstetrics is under particular scrutiny because it pays amongst the highest amount in litigation settlements of any clinical discipline [1, 2]. Evidence suggests that physicians perceived as being at higher liability risks are likely to practice “*defensive*” medicine, whereby avoidance of litigation may take precedence over the patient’s best interests in medical decision-making [1]. In obstetrics, this approach could lead to an increase in unnecessary procedures, such as unwarranted cesarean sections [3], electronic fetal monitoring, epidural analgesia [4], and fetal scalp blood sampling [5] during labor.

It is important to ensure that a well-balanced, strategic approach is taken for medical and obstetrical malpractice reform so that the control of malpractice litigation costs is accompanied by fair compensation of patients injured due to medical negligence [1]. Such an approach requires the careful analysis of policies that exist globally and their short-term and long-term consequences [6, 7], taking into account the influence of multiple stakeholders with different interests, including patients, clinicians, healthcare managers, and policy makers [8].

Efforts have been made to address medical malpractice, patient safety [9–11], and litigation costs including a specialized court system to review cases, financial support or compensation to victims, and strategies to facilitate communication between the physician and patient outside the court-room setting. However, most of the work to date has evaluated this problem in the USA, which has a different healthcare and economic structure than that of low- and middle-income economy countries (LMICs); therefore, the results might not be generalizable to all settings. A synthesis of policies that exist globally may provide clarity to this issue. As such, we aimed to synthesize evaluative or comparative reports assessing medical liability reforms and quality improvement strategies to control litigation costs of medical errors in obstetrics.

## Methods

A rapid scoping review was conducted to synthesize our current knowledge of medical liability reforms and quality improvement strategies to control medical errors and avoidable adverse effects associated with obstetrical procedures. A rapid review is a form of knowledge synthesis in which components of the systematic review process are simplified or omitted to produce information quickly [12]. Scoping reviews are meant to provide users and researchers with an overview of a topic so as to identify key concepts, knowledge gaps, and types of evidence within an evolving field of research [13, 14]. We were commissioned to conduct this rapid scoping review by the World Health Organization (WHO) South Africa within a 6-week timeline. We used the PRISMA statement to report the results of our review (Additional file 1) [15].

### Protocol

A protocol was developed and revised using feedback from the research team, including clinicians, review methodologists, and members of the WHO country office in South Africa. The final protocol can be found in Additional file 2. We did not register our protocol because we had limited time.

### Information sources and literature search

To identify potentially relevant documents, the following bibliographic databases were searched from 2004 to June 2015: MEDLINE, EMBASE, LexisNexis Academic, the Legal Scholarship Network, Justis, LegalTrac, QuickLaw, and HeinOnline. The search strategies were drafted by an experienced librarian (Dr. McGowan) and further refined through team discussion. The final search strategy for MEDLINE can be found in Additional file 3. The final search results were exported into EndNote, and duplicates were removed by a library technician. The electronic database search was supplemented by searching the Canadian Medical Protective Association website (https://www.cmpa-acpm.ca/en) and scanning relevant reviews.

### Eligibility criteria

The eligibility criteria were defined using the Population, Intervention, Comparator, Outcome (PICO) framework (Table 1) [16]. The PICO framework can be used to construct research questions, which is common practice in systematic reviews. The well-constructed research question allows for the correct definition of which evidence is needed to answer the research question, maximizes the recovery of evidence in literature databases, and focuses the scope of the literature search, reducing unnecessary searching [17].

**Table 1:** Eligibility criteria for reports

*P* (*population*)	Women who had an obstetrical procedure and experienced an adverse event and/or a medical error
	Any type of claim related to obstetrical care
*I* (*intervention*)	Medical liability reforms and quality improvement strategies, such as reforms, tort reforms, damage award limits, frivolous suit penalties, expert witness requirements, statutes of limitations, immunity provisions, and no-fault compensations
	Patient safety initiatives were included if they were policies/models implemented at the population level (e.g., country-wide, state-wide)
*C* (*comparator*)	Other policy reforms/models/frameworks
	No policy or no comparator
*O* (*outcome*)^a^	Litigation costs
	Cost containment

^a^Selected by the commissioners of this review

All types of study designs and reviews evaluating or comparing different policies were included. The publication date was limited from 2004 to 2015, to include current evidence. We also limited our rapid scoping review to published documents written in the English language, to optimize feasibility.

### Reports selection

All levels of screening were performed using our online tool, Synthesi.SR (http://www.breakthroughkt.ca). Our online tool enables quick screening of titles, abstracts and full-text articles, captures reasons for exclusion, and allows the identification and resolution of disagreements between reviewers [18]. The screening criteria were established a priori and calibrated through a training exercise performed by 9 reviewers (ACT, RC, VN, JA, CW, HM, KW, MK, RW) on a random sample of 50 titles and abstracts. After reaching 90% agreement, pairs of reviewers screened the literature search results independently. Full-text articles of relevant titles and abstracts were retrieved for further review and were screened by pairs of independent reviewers (RC, VN, JA, CW, HM, KW, MK, RW, WZ) after 95% agreement was reached on the training exercise using a random sample of 15 full-text articles. Overall, we reached 87% agreement between reviewers at level 1 (title and abstract) and level 2 (full-text) screening. Discrepancies between pairs of reviewers were resolved through discussion or a third adjudicator (ACT).

### Data abstraction and data items

A standardized data abstraction form was developed a priori and pilot-tested by nine team members independently on a random sample of three articles and revised iteratively by the project team. After reaching 85% agreement, pairs of reviewers (RC, VN, JA, CW, HM, KW, MK, RW, WZ) independently read each document and extracted relevant data using the standardized data abstraction form. Discrepancies were resolved by a third reviewer to ensure correctness and consistency of all the data (RC, WZ). The extracted data included report characteristics (e.g., first author, year of publication, publication type, report design), information related to reforms to control damages and financial liabilities, as well as advantages/limitations of the strategies according to the authors’ perspectives.

### Methodological quality (or risk of bias) appraisal

As per guidance on the conduct of scoping reviews [19], we did not appraise methodological quality of the included reports, which is consistent with scoping reviews on health-related topics [19, 20].

### Synthesis

The findings of this review are presented descriptively. The medical liability reforms and quality improvement strategies to control damages and financial liabilities identified are presented in tables and categorized by type of strategy and country of origin for the policy. Definitons of terms can be found in Additional files 4 and 5.

## Results

**Fig. 1:**
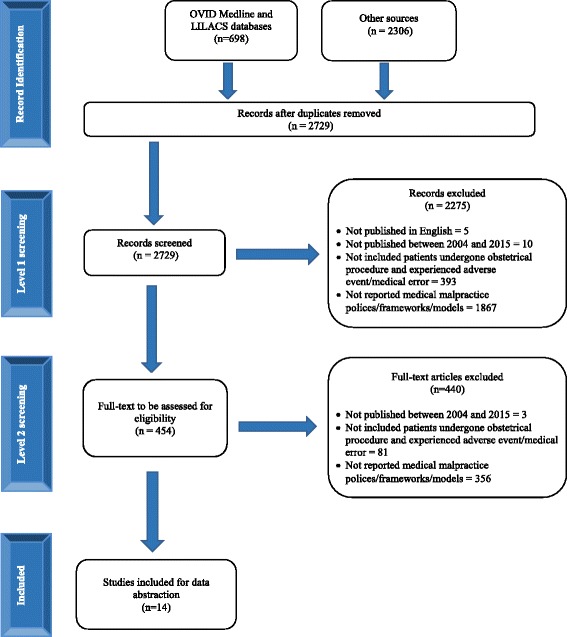
Study flow chart

The literature search yielded a total of 2729 citations (Fig. 1). Upon completion of title and abstract screening, 454 full-texts were deemed potentially relevant and reviewed. Subsequently, 14 documents fulfilled our eligibility criteria and were included [3, 21–33].

### Report characteristics

Most publications were research studies. Eight cohort studies [23–25, 27, 28, 30, 32, 33], four uncontrolled before-after reports [3, 21, 29, 31], and two narrative reviews [22, 26] were included. The reports were published between 2004 and 2015, and the majority (86%) were conducted in the USA [3, 21–26, 29–33] (Table 2). The majority of the documents included a small sample size (Table 3). The duration of follow-up for the included reports ranged from 2 to 17 years across eight reports and was not reported in six reports [3, 21–23, 27–29, 31] (Table 3).

**Table 2:** Report characteristics

Item (*n* = 14)		Count	Percent (%)
Year of publication	2004–2007	5	36
	2008–2011	5	36
	2012–2015	4	29
Country of publication	USA	12	86
	Canada	1	7
	UK	1	7
Document type	Research study	12	86
	Review of models	2	14
Strategy type^a^	No-fault approach	2	12
	Patient safety initiatives	4	24
	Communication and resolution	2	12
	Caps of compensation and attorney fees	6	35
	Alternative payment system and liabilities	2	12
	Limitations on litigation	1	6

^a^Some documents reported on more than one strategy

**Table 3:** Strategies to mitigate litigation characteristics

Author, year (setting)	Sample	Strategy name	Follow-up period	Adverse events
Behrens 2011 [21] (Mississippi, USA)	4878 lawsuits	Mississippi tort reform legislation	7 years	Obstetrical/gynecological
Bovbjerg 2005 [22] (USA)	226 claims + 281 surveys	Administrative compensation model	8 years in Florida, 9 years in Virginia	Birth-related neurologic injuries
Currie 2008 [30] (USA)	NR	Noneconomic damage caps	NR	Obstetrics
		Limit joint and several liability		
		Caps on punitive damages		
		Reforms of the collateral source rule		
Edwards 2010 [23] (Virginia, USA)	571 obstetricians	Virginia Birth-Related Neurological Injury Compensation Program (BIP)	19 years	Obstetrics
Ho 2011 [24] (USA)	234,826 adverse events	Apology laws	NR	Any medical malpractice including obstetrics
Iizuka 2013 [25] (USA)	NR	Caps on noneconomic damages (CapsNED)	NR	Birth trauma injury to neonate; obstetric trauma to mother
		Caps on punitive damages (CapsPD)		
		Collateral source rule (CSR) reform		
		Joint and several liability reform		
Kachalia 2010 [31] (Michigan, USA)	1131 claims	Medical Error Disclosure Program	4 years	Any medical error
Kilgore 2006 [32] (USA)	NR	Damage caps strategy	NR	Internal medicine, surgery, obstetrics
		Statute of limitations		
Milne 2013 [27] (Canada)	39 participating hospitals	Managing Obstetrical Risk Efficiently (MORE)	8 years (costs) & 9 years (catastrophic infant claims)	Obstetrics
Pegalis 2012 [26] (USA)	NA	Patient safety guidelines	NR	Obstetrics
Santos 2015 [29] (USA)	5 labor and delivery sites	Risk reduction labor and delivery model	2 years and 3 months	Obstetrics; labor and delivery events (shoulder dystocia and fetal distress)
Studdert 2004 [3] (California, USA)	152 cases	California’s Medical Injury Compensation Reform Act (MICRA) cap	17 years	Surgical errors, obstetrical errors, missed or delayed diagnoses, and drug errors
Thorpe 2004 [33] (USA)	NR	Award cap strategy	NR	Obstetrics, surgical, internal
Winn 2007 [28] (UK)	7352 obstetrics and gynecology claims	Clinical Negligence Scheme for Trusts	3 years	Any medical malpractice obstetrics

*Abbreviations*: *NA* not applicable, *NR* not reported

### Strategies for improving medical malpractice and liability systems

Several initiatives for improving medical malpractice were reported and are summarized below according to the type of strategy and country of origin (Table 3 and Additional file 6). A detailed description of all strategies, including how strategies were evaluated or compared, as well as advantages, limitations, and cost savings of these strategies can be found in Table 3 and Additional files 6, 7, 8, and 9.

### No-fault approach

#### USA

In a review, Bovbjerg [22] examined programs enacted in Virginia and Florida using administrative closed malpractice claims data as well as patient and physician surveys. These programs kept obstetric liability coverage available and decreased tort premiums. Administrative claims were perceived to be lower than expected (196 over 8 years in Florida, and 30 over 9 years in Virginia) and perceived to be affordable. However, it was not possible to determine the impact of these programs on patient safety due to the small number of cases.

Similarly, Edwards [23] reported that Virginia’s no-fault approach, entitled the Birth Injury Program (BIP), shielded participating physicians from the negative effects of malpractice claims for selected injuries. They also found that when controlling for variables that influenced cesarean rates, participating physicians had lower adjusted cesarean rates than nonparticipating physicians. Specifically, participation was associated with a 0.80% (95% confidence interval, CI − 0.23–0.01) decrease in the adjusted cesarean rate. The results of this cohort study support the theory that BIP encourages fewer cesarean; however, the results were not statistically significant. The program covered malpractice claims due to birth-related neurological injuries resulting from oxygen deprivation, mechanical injuries occurring during labor and delivery, or resuscitation immediately post-delivery in the hospital.

### Patient safety policy initiatives

#### USA

In a review, Pegalis and Bal [26] examined whether safety guidelines originating from past medical malpractice litigation practices could lower costs associated with medical liability. In anesthesia and obstetrics, after the implementation of safety measures, the authors reported a decrease in the incidence and costs associated with medical malpractice. Specifically, anesthesia-related deaths decreased from one per 10,000 anesthetic procedures to one per 200,000 procedures. Also, improved perinatal outcomes were reported with lower maternity and fetal injury rates, primary cesarean delivery rates, and litigation rates. The average compensation payment decreased from more than $27 million to approximately $2.5 million per year, and serious adverse events such as death and permanent or severe temporary harm were reduced from five events per year to zero events. However, no data were presented to show statistical significance of these findings.

Santos et al. [29] evaluated a risk reduction model for labor and delivery through an uncontrolled before-after study. This multilevel integrated practice and coordinated communication model consisted of four key components: standardized practice guidelines, process documentation, event reporting and disclosure policy, and audit and feedback. Medical liability risk and administrative data sets were analyzed. Over 2 years after implementation, reporting of unintended events increased significantly (43 vs. 84 per 1000 births, *p* < 0.01) while high-risk malpractice events decreased significantly (14 vs. 7 per 1000 births, *p* < 0.01).

#### Canada

Milne et al. [27] performed a cohort study to assess the impact of the Managing Obstetrical Risk Efficiently (MORE) program at Canadian Hospitals after 10 years of its implementation. The program consisted of three educational modules targeting health care professionals: (1) learning together, (2) working together, and (3) changing culture. Survey results from 174 Canadian hospitals as well as claims data from 39 hospitals insured for liability by the Healthcare Insurance Reciprocal of Canada (HIROC) were analyzed. A significant reduction (*p* < 0.01) was shown in average costs acquired in the labor and delivery units after the program. Incurred costs included payments for the HIROC lawyer, adjuster, expert opinion, settlements paid to claimants, claimant legal costs, and the reserve in Ultimate Probable Cost (an estimation of the cost of the claim through to its final disposition). Analysis of the catastrophic infant claims data showed an average reduction of two infant claims per year over a period of 8 years (2002–2010).

#### UK

Winn [28] described strategies consisting of a range of reactive and proactive risk management systems and processes. They examined whether the risk management standards had an effect on clinical negligence claims in a cohort of maternity services. The percentage of obstetrics and gynecology claims decreased from 28% in 1995–1996 to 16% in 2005–2006. However, there was no evidence that the standards had a direct impact on the number, severity, or type of claims.

### Communication and resolution

#### USA

Ho and Liu [24] focused on the impact of apology laws in dealing with medical malpractice litigations in a cohort of 36 states that enacted various forms of apology laws. These laws stated that apologies made by medical practitioners cannot be used as evidence in medical malpractice litigation. The authors analyzed data from the National Practitioner Data Bank, which contains information on all malpractice cases with payment made by medical practitioners in the USA since 1991. They concluded that apologies were most relevant for cases involving obstetrics and anesthesia, infants, inappropriate management by a physician, as well as failures to diagnose conditions. The authors found that in states with an apology law, payments to plaintiffs in malpractice cases were $32,342 [13% (*p* < 0.01)] less than to plaintiffs in states without such a law. They also found that the impact of apologies differed considerably by allegation nature, with apologies for obstetric-related claims having the highest value at $125,000, followed by anesthesia at $87,000.

Kachalia et al. [31] compared liability claims and costs before and after implementation of the University of Michigan Health System (UMHS) program that focused on a comprehensive claims management model of disclosure with an offer of compensation for harmful medical errors. In this uncontrolled before-after study, the authors found that, after program implementation, the average monthly rate of new claims declined from 7.03 to 4.52 per 100,000 patient encounters (RR 0.64; 95% CI 0.44–0.95) and average monthly rate of lawsuits dropped from 2.13 to 0.75 per 100,000 patient encounters (RR 0.35; 95% CI 0.22–0.58). As well, the median time from claim reporting to resolution decreased from 1.36 to 0.95 years, average monthly cost rates decreased for total liability (RR 0.41, 95% CI 0.26–0.66), as did patient compensation (RR 0.41, 95% CI 0.26–0.67), and non-compensation-related legal costs (RR 0.39, 95% CI 0.22–0.67). However, the authors noted that prior to the inception of the UMHS disclosure program, the state of Michigan had a multicomponent malpractice reform (i.e., damage caps, 6-month compulsory pre-suit notice and a new expert witness foundation) in place since 1994, which may have contributed to the decrease in liability claims and costs.

### Caps on compensation and attorney fees

#### USA

Three reports [3, 21, 33] reviewed state-level experiences with award caps on noneconomic (i.e., damages in medical malpractice cases for non-pecuniary harms such as pain and suffering) and punitive damages. Behrens [21] explored Mississippi’s tort reform legislation implementation in an uncontrolled before-after study comparing data regarding lawsuits against physicians insured by Medical Assurance Company of Mississippi (MACM) and MACM-insured obstetrician–gynecologists from 1986 to 2010. With a $500,000 limit on noneconomic damages, medical liability insurance premiums for MACM-insured physicians were both lowered and reimbursed. The study showed that during the pre-implementation period (2000–2002) MACM-insured obstetrician–gynecologists experienced an average of 60 lawsuits per year. During the implementation period (2003–2004), the number of lawsuits dropped to an average of 20 per year. In the 5-year period (2005–2009) after the implementation of tort reform, there were 15 lawsuits per year against MACM-insured obstetrician–gynecologists. However, the authors stated that although MACM’s risk management department was intensively involved in risk management with its insured physicians, their reports have insufficient data showing that risk management or safety measures accounted for the decrease in frequency of claims and lawsuits after the implementation of tort reforms in Mississippi.

Thorpe [33] estimated the impact of award caps on malpractice insurance premiums in a cohort of 24 states between 1985 and 2001 using data from the National Association of Insurance Commissioners Loss ratios by medical malpractice carriers in states that capped malpractice awards were 12% lower than in states without these caps. In addition, loss ratios were 13% lower in states with discretionary collateral offsets (a rule that states that a complainant could recover the full amount of the reward even if the complainant received money from other sources). Loss ratios were 25% lower in states that adopted award caps and discretionary collateral offset reforms. Notably, only states with discretionary collateral offsets had lower medical insurance premiums or improved malpractice firms profits; states with mandatory offsets showed no impact on loss ratios. Physicians’ malpractice insurance premiums in states with a cap on awards were 17% lower than in states without such caps. The analysis found no association between the adoption of other state tort reforms (i.e., punitive damage cap, mandatory collateral offset rule, attorney fee caps) on loss ratios, premiums, joint liability, caps on attorneys’ fees, or discretionary collateral offsets during the same time frame.

Studdert et al. [34] analyzed awards before and after they were subjected to the California’s Medical Injury Compensation Reform Act (MICRA) using a sample of jury verdicts in California that were subjected to the State’s $250,000 cap on noneconomic damages. The severity of each injury was scored using the National Association of Insurance Commissioners (NAIC) nine-point scale (e.g., emotional injury only (score = 1) to death (score = 9)). In this uncontrolled before-after study, the absolute reduction in noneconomic damages under the cap for grave injury (NAIC score = 8) was seven times larger than that for minor injury. Proportional reductions in noneconomic damages under the cap ranged from 2 to 82% (mean 37%; standard deviation (SD) ± 20%) for nonfatal injuries and from 6 to 88% (mean 47%; SD ± 24%) for fatal injuries. Severe neurological injuries to newborns (9/20) accounted for the smallest percentage of reduction in noneconomic damages. Injuries that caused pain or disfigurement but not significant loss of physical functioning (12/20) had the largest percentage of reduction in noneconomic damages under the cap. Authors concluded that the fiscal impact on verdicts was distributed inequitably across different types of injuries. However, they reported that the data collection methods might have biased reports toward larger verdicts, as their data set did not include all plaintiff verdicts in California (i.e., injured patients who never sought compensation or plaintiffs with unresolved verdicts were excluded). Also, the analysis assumed that juries were ignorant of the cap, which may not be accurate.

### Multicomponent models

#### USA

#### Caps on compensation and attorney fees & alternative payment system and liabilities

Iizuka [25] performed a cohort study using data from the Nationwide Inpatient Sample created by the Agency for Healthcare Research and Quality (1994–2007) that contained hospital discharge data for individual patients. They found mixed results for caps on noneconomic damages. Joint and several liability reform (which occurs when a claimant may pursue an obligation against any one party as if they were jointly liable and it becomes the responsibility of the defendants to sort out their respective proportions of liability and payment) increased doctor’s accountability, reducing preventable medical complications. However, collateral source rule reform (which occurs when a rule that prohibits the admission of evidence that the plaintiff or victim has received compensation from some source other than the damages sought against the defendant) and caps on punitive damages were found to increase medical complications.

Another cohort study explored whether reforms––caps on punitive damages, influenced the types of procedures performed and the health outcomes of mothers and their infants. Currie and MacLeod [30] analyzed data from the national vital statistics natality files from 1989 to 2001. The study focused on four reforms: caps on punitive damages, caps on noneconomic damages (pain and suffering), reform of the rule of joint and several liability (this makes each defendant in a tort lawsuit liable for the entire amount of plaintiff’s damages, also called the deep pocket rule), and reforms of the collateral source rule. The authors concluded that caps on damages increased the occurence of cesarean sections by about 5% and preventable complications of labor by 6%. They found no effects of tort law changes on the probability of low appearance, pulse, grimace, activity, and respiration (APGAR) scores for the newborn. The joint and several liability reform reduced cesarean sections by about 7% and preventable complications of labor by 13%.

#### Caps on compensation and attorney fees & limitations on litigation

Kilgore et al. [32] estimated the effect of changes in tort law on medical premiums between 1991 and 2004 for three specialties: obstetrics/gynecology, general surgery, and internal medicine. They estimated a “dose-response” effect for inflation-adjusted values of damage caps and the effect of investment returns on malpractice premiums using the Medical Liability Monitor annual survey of physician insurers. Specifically in the field of obstetrics/gynecology, this cohort study found that damage caps lowered malpractice insurance premiums by 26% (*p* < 0.01). Moreover, an increase of $100,000 in the statutory cap on noneconomic damages increased malpractice premiums by 4% (*p* < 0.01). The data showed that the presence of a statute of limitations on malpractice suits reduced premiums, while the length of the statute was associated with a statistically significant increase in malpractice insurance premiums (e.g., longer statutes of were associated with higher premiums). However, these findings may be prone to bias due to small sample size. Also, the authors did not explore potential confounders, such as limits on attorney fees and pre-trial screening procedures.

## Discussion

### Main findings

Despite the reported high costs associated with medical malpractice litigation, very few documents evaluated models to reduce litigation. We identified 14 documents that evaluated medical liability reforms and quality improvement strategies [3, 21–33]. Most of the literature is from the USA, which is likely because of the larger number of medical malpractice claims that occur per year in that country relative to other countries [35]. None of the reports originated from LMICs.

Various initiatives for improving the medical malpractice litigation system were found, including no-fault approaches, patient safety initiatives, communication and resolution, caps on compensation and attorney fees, alternative payment system and liabilities, and limitations on litigation and multicomponent models.

Our review found that initiatives to reduce medical malpractice litigation could be associated with a decrease in avoidable adverse and malpractice events. For example, communication and resolution initiatives to optimize patient safety were explored by Ho and Liu [24] and Kachalia et al. [31]. They found that these initiatives have the potential to decrease claims and liability costs and to reduce injuries as a result of patient safety efforts. No-fault approach initiatives were examined by Bovbjerg [22] and Edwards [23]. These authors found that the administrative compensation model kept obstetrical liability coverage available and decreased tort premiums; Edwards [23] showed that Virginia’s no-fault approach protected participating physicians almost entirely from the negative effects of malpractice claims for certain injuries. Caps on compensation and attorney fees showed benefits related to the resolution of some claims more easily [21]; and reductions in obstetrics/gynecology premiums [32], frequency of claims, frequency of lawsuits [21], and loss ratios [33]. However, some disadvantages of these strategies were also reported including the association with increase in medical errors [25], as well as the incidence of cesarean sections and preventable complications [30]. Initiatives to reduce the burden of liability pressures and financial burden of claims payments [25, 30] were also investigated. These initiatives showed an association with the reduction in cesarean sections and preventable complications of labor [30] and also medical errors [25]. Only one report [32] reported on limitations of litigation. The authors found that the presence of statute of limitations reduced premiums and length of statute was associated with a statistically significant increase in premiums. However, due to heterogeneous settings (e.g., economic structure, healthcare system) and variation in the outcomes reported, the advantages and disadvantages of initiatives may vary.

There are some limitations to our rapid scoping review methods. First, scoping reviews have inherent limitations because the focus is to identify knowledge gaps, inform future research, and identify implications for decision-making [20]. As such, we did not appraise the methodological quality of the included reports; hence, we are unable to formally comment on their scientific rigor. To produce the review in a timely manner, we simplified some of the components of the scoping review process. For example, the final protocol was not registered. We only included documents that were published after 2004 and written in the English language. References lists of relevant documents were not scanned. We were unable to contact authors for further potentially relevant reports due to a lack of time. In addition, we included reports published from 2004 to 2015; however, some of these reports used sampling frames from before this period. As well, all included reports were from developed countries and most were from the USA. This might be because we limited the included documents to those in English or because such medical malpractice policies have not been fully examined in LMIC settings. Furthermore, we focused inclusion on documents that specifically discussed medical malpractice in the obstetric field. As such, medical malpractice models that might be relevant to obstetrics, but have not explicitly described malpractice risks in obstetrics may have been excluded, as this was not the focus of our research. We did not consider specific legislative and regulatory frameworks of the countries, which could potentially impact the effectiveness of the policies examined here, as this was outside the scope of this review. In addition, we did not include other outcomes, such as malpractice burden on provider availability, access to care, and specific obstetric outcomes (e.g., operative vaginal delivery), as they were of less interest to the commissioners of this review.

Although we did not formally appraise the methodological quality of the included reports, there are some limitations worth noting. For example, during the study conduct, changes to multiple factors, such as the law, data monitoring, medical practice, and potentially unknown factors may have occurred, rendering definitive conclusions about the effectiveness of the policy unattainable. In addition, some of the reports were small and had insufficient power [22]. All of the included documents were retrospective in nature and a high-quality, empirical, prospective documents on this topic was not identified. Prospective studies such as randomized trials might be difficult to conduct in this area. However, researchers could consider interrupted time series designs or controlled before and after studies that use other states or countries as comparators. Retrospective studies could similarly be improved by measuring potential confounders. And, all studies should consider including outcomes of importance to knowledge users such as costs and patient safety.

## Conclusions

In conclusion, despite the reported high costs associated with medical malpractice litigation specifically in the obstetrics medical specialty, only a few medical malpractice models were evaluated or compared. Included reports showed that initiatives to reduce medical malpractice litigation could be associated with a decrease in adverse and malpractice events. However, due to heterogeneous settings (e.g., economic structure, healthcare system) and variation in the outcomes reported, the advantages and disadvantages of initiatives may vary. We suggest that any policy that is implemented be evaluated for effectiveness using a rigorous study design, as well as cost through an economic analysis, and provide details surrounding the context where policy will be implemented.

## Additional files

Additional file 1: PRISMA Checklist. (DOCX 30 kb)

Additional file 2: Protocol. (DOCX 42 kb)

Additional file 3: Search strategy. (DOCX 20 kb)

Additional file 4: Terminology. (DOCX 20 kb)

Additional file 5: Costs–descriptions by authors of included reports. (DOCX 34 kb)

Additional file 6: Description of the strategies to mitigate litigation. (DOCX 42 kb)

Additional file 7: Initiatives for improving mdical malpractice definitions. (DOCX 17 kb)

Additional file 8: Evaluation of strategies to reduce litigation. (DOCX 45 kb)

Additional file 9: Outcomes of the strategies to reduce litigation. (DOCX 48 kb)

## Abbreviations

APGAR: Appearance, pulse, grimace, activity, and respiration
BIP: Birth Injury Program
CI: Confidence interval
HIROC: Healthcare Insurance Reciprocal of Canada
LMIC: Low- and middle-income economy country
MACM: Medical Assurance Company of Mississippi
MICRA: Medical Injury Compensation Reform Act
MORE: Managing Obstetrical Risk Efficiently
NAIC: National Association of Insurance Commissioners
UMHS: University of Michigan Health System
WHO: World Health Organization
