# Supplementary material for: Evaluative reports on medical malpractice policies in obstetrics: a rapid scoping review
Source: Syst Rev. 2017 Sep 6;6:181. doi: 10.1186/s13643-017-0569-5 (PMC5586050; doi:10.1186/s13643-017-0569-5)
Supplement: Supplementary file 2 — Protocol. (DOCX 42 kb) [file 13643_2017_569_MOESM2_ESM.docx]

Additional File 2. Protocol

# Background and Objective

There is general agreement in the medical community that the current medical malpractice system is becoming costly and inefficient [[1](#_ENREF_1)]. Litigation costs can range from 2.4% to 10% of healthcare spending, while unnecessary tests and procedures add further to the healthcare expenditure [[1](#_ENREF_1)]. The collapsing state of the medical malpractice system is underpinning the belief within healthcare that malpractice litigation “has long since surpassed sensible levels and that major tort reform is overdue” [[2](#_ENREF_2)].

The clinical specialty of obstetrics is under particular scrutiny for paying amongst the highest litigation settlements [[1](#_ENREF_1), [3](#_ENREF_3)]. Evidence suggest that physician specialists perceived as being under higher liability risks are likely to practice ‘*defensive*’ medicine, whereby their practices are not solely focused on patient’s health, but also on safeguarding against possible medical malpractice liability [[1](#_ENREF_1)]. In obstetrics, this approach could lead to the potential increase in unnecessary clinical procedures, such as unwarranted cesarean sections [[2](#_ENREF_2)]. This implies that choices and options for obstetrical care could be, to some extent, “held hostage by the fear of lawsuits” [[4](#_ENREF_4)]. In addition, increased liability could partly influence high levels of dissatisfaction among physicians specializing in obstetrics and are thought to be a driving factor for early retirement, contributing to the shortage of these specialty physicians [[5](#_ENREF_5)].

It is important to make sure that we take a well-balanced, strategic approach to medical and obstetrical malpractice reforms so that the control of malpractice litigation costs is accompanied with the fair compensation of patients injured by medical negligence [[1](#_ENREF_1)]. Such an approach requires the careful analysis of world-wide policies and their short-term and long-term consequences [[6](#_ENREF_6), [7](#_ENREF_7)], taking into account the presence of multiple stakeholders including patients, clinicians, healthcare managers, and policy makers who have conflicting interests [[8](#_ENREF_8)].

The objective of our study was to complete a rapid scoping review to map all available evidence in the literature regarding medical malpractice models/frameworks/policies to control damages and financial liabilities in obstetrics in any country (including high income-economy countries [HIC] and low-to-middle income economy countries [LMIC]).

# Methods

## Scoping reviews

A scoping review aims to “map the literature on a particular topic or research area and provide an opportunity to identify key concepts, gaps in the research; and types and sources of evidence to inform practice, policymaking, and research” [[9](#_ENREF_9)]. A scoping review essentially follows the same steps of a systematic review recommended by the Cochrane Collaboration [[10](#_ENREF_10)], except the quality of included reports is not appraised because the purpose is to map out the literature and identify areas to conduct future systematic reviews.

## Rapid reviews

Rapid reviews are a form of knowledge synthesis in which components of the systematic review process are simplified or omitted to produce information in a timely manner [[11](#_ENREF_11)]. Depending on the scope and timelines, rapid reviews will streamline some of the processes recommended by the Cochrane Collaboration, such as only 1 reviewer screening the literature search results, abstracting data, and appraising quality. A meta-analysis generally is not conducted for a rapid review.

## Search Strategy

We will use the methodologically rigorous rapid scoping review approach as described in the Cochrane Handbook for Systematic Reviews. We will conduct a systematic search across the following electronic databases from inception onwards: MEDLINE (OVID interface), EMBASE (OVID interface), LexisNexis Academic, and the Legal Scholarship Network. The general search terms included those related to medical malpractice, statutes of limitation, financial limitation, limited damages, impact on damages and restricted statues; however the search is limited to publications in English from 2004 onwards.

A search conducted on May 15, 2015 of MEDLINE using the defined terms retrieved approximately 380 citations. It is anticipated that approximately 1400 citations will result after searching EMBASE, the Cochrane Library, and legal databases in addition to MEDLINE. We will further refine the search strategy iteratively with input from the investigators and in consultation with our experienced information specialist. The search strategy will be peer reviewed by another librarian using the Peer Review of Electronic Search Strategies (PRESS) checklist [[12](#_ENREF_12)]. After this exercise, the search strategy will be amended, as required. The information specialist will execute all final searches, export the results into EndNote, and remove all duplicates from the search results. The results will then be uploaded to Synthesi.SR [[13](#_ENREF_13)], proprietary software available through the Li Ka Shing Knowledge Institute of St. Michael’s Hospital.

## Study Selection: Screening

Prior to commencing the screening process, a calibration exercise will be conducted to ensure reliability in correctly selecting articles for inclusion. This will entail screening a random sample of 5% of the included citations by all team members, independently. Eligibility criteria will be modified if low agreement is observed between the reviewers (e.g., percent agreement <90%). Two reviewers will then independently screen the remainder of the search results for inclusion using a pre-defined relevance criteria form for all levels of screening (e.g., title and abstract, full-text review). Discrepancies will be resolved by discussion or the involvement of a third reviewer.

## Data Abstraction:

A data abstraction form will be drafted and pilot-tested by all team members independently on a random sample of 10 articles and revised iteratively by the study team while the search is completed. It is anticipated that the data items will include information related to reforms to control damages and financial liabilities. Pairs of team members will independently read each article and extract the relevant data. Differences in abstraction will be resolved by discussion or the involvement of a third reviewer.

## Synthesis

We will narratively describe the reports included. The models/frameworks/policies/reforms to control damages and financial liabilities identified will be presented in tables and categorized by type of strategy, obstetrical issue, and country of origin for the policy.

# References

1. Medical Malpractice Reform. Health Cost Containment and Efficiencies: NCSL Briefs for State Legislators [Internet]. 2011.[71-4 pp.]. Available from: <http://www.ncsl.org/documents/health/IntroandBriefsCC-16.pdf>. Accessed March 2016.

2. Studdert DM, Mello MM, Brennan TA. Medical malpractice. N Engl J Med. 2004;350(3):283-92.

3. Knox GE, Simpson KR, Garite TJ. High reliability perinatal units: an approach to the prevention of patient injury and medical malpractice claims. J Healthc Risk Manag. 1999;19(2):24-32.

4. Schifrin BS, Cohen WR. The effect of malpractice claims on the use of caesarean section. Best Pract Res Clin Obstet Gynaecol. 2013;27(2):269-83.

5. Anderson BL, Hale RW, Salsberg E, Schulkin J. Outlook for the future of the obstetrician-gynecologist workforce. Am J Obstet Gynecol. 2008;199(1):88 e1-8.

6. Medical liability practices in Canada: towards the right balance. Task Force on Adverse Health Events Background Volume III Submissions [Internet]. 2005. 3:[29-74 pp.]. Available from: <http://www.gov.nl.ca/ahe/submissions/03_CanadianMedicalProtectiveAssociation.pdf>. Accessed March 2016.

7. Cortez N. A medical malpractice model for developing countries? Drexel Law Review. 2011;4:217-41.

8. Chervenak FA, McCullough LB. Neglected ethical dimensions of the professional liability crisis. Am J Obstet Gynecol. 2004;190(5):1198-200.

9. Daudt HM, Van Mossel C, Scott SJ. Enhancing the scoping study methodology: a large, inter-professional team’s experience with Arksey and O’Malley’s framework. BMC Med Res Methodol. 2013;13(1):1.

10. Higgins J, Green S, (editors). Cochrane Handbook for Systematic Reviews of Interventions Version 5.1.0: The Cochrane Collaboration; 2011 [updated March 2011]. Available from: [www.cochrane-handbook.org](http://www.cochrane-handbook.org) .

11. Ganann R, Ciliska D, Thomas H. Expediting systematic reviews: methods and implications of rapid reviews. Implementation Science. 2010;5(1):1.

12. McGowan J, Sampson M, Salzwedel DM, Cogo E, Foerster V, Lefebvre C. PRESS Peer Review of Electronic Search Strategies: 2015 Guideline Statement. J Clin Epidemiol. 2016;75:40-6.

13. Knowledge Translation Program.(editors). Synthesi.SR. Toronto, Ontario. Li Ka Shing Knowledge Institute, St. Michael's Hospital. 2014. <http://www.breakthroughkt.ca/login.php>. September 2015.
