# Supplementary material for: Evaluative reports on medical malpractice policies in obstetrics: a rapid scoping review
Source: Syst Rev. 2017 Sep 6;6:181. doi: 10.1186/s13643-017-0569-5 (PMC5586050; doi:10.1186/s13643-017-0569-5)
Supplement: Supplementary file 3 — Search strategy. (DOCX 20 kb) [file 13643_2017_569_MOESM3_ESM.docx]

**Additional File 3. Search strategy**

**WHO Malpractice – Medline Search Strategy (Literature Search performed: June 15, 2015)**

1. Obstetrics/

2. "Obstetrics and Gynecology Department, Hospital"/

3. exp Obstetric Surgical Procedures/

4. obstetric$.tw,hw.

5. exp Obstetric Labor Complications/

6. exp "Dilatation and Curettage"/

7. exp Hysterectomy/

8. Sterilization, Tubal/

9. Salpingostomy/

10. exp Pregnancy Complications/

11. cerebral palsy/

12. Asphyxia Neonatorum/

13. (abortion$ or cervical cerclage or colpotomy or culdoscop$ or fetoscop$ or hysteroscop$ or hysterotomy).tw.

14. (paracervical block$ or obstetric$ anesthe$ or obstetric$ anaesthe$).tw.

15. (Cesarean or Episiotom$ or obstetric$ abstraction$ or fetal version).tw.

16. ((induc$ or augmentation or premature or pre-term or preterm or obstructed) adj (labour or labor)).tw.

17. (Abruptio Placentae or breech or Cephalopelvic Disproportion or premature rupture of fetal membrane$ or prom or fetal membranes premature rupture or Dystocia or Uterine Inertia or Chorioamnionitis or Placenta Accreta or Placenta Previa or Postpartum Hemorrhage or Uterine Inversion or Uterine Rupture or Vasa Previa).tw.

18. (Fetal Death or Fetal Resorption or Stillbirth or perinatal death or peri-natal death or Maternal Death or Birth Injuri$ or obstetric$ paralys$).tw.

19. (pre-eclampsia or dilatation or Curettage or Vacuum aspiration).tw.

20. (asphyxia neonatorum or cerebral palsy or birth asphyxia or fetal pulmonary embolism or dystocia).tw.

21. exp Dystocia/ or exp Pregnancy Complications, Cardiovascular/

22. or/1-21

23. exp Medical Errors/

24. ae.fs.

25. (error$ or advers$ or mistake$ or negligence).tw.

26. or/23-25

27. 22 and 26

28. exp Malpractice/

29. Expert Testimony/

30. (reforms or tort reform$ or damage award limit$ or lawsuit$ or immunity provision$).tw.

31. (immunity provision$ or immunity clause$ or fault compensation or Malpractice or expert witness$).tw.

32. (statutes adj2 limitations).tw.

33. lj.fs.

34. exp Jurisprudence/

35. or/28-34

36. 27 and 35

37. limit 36 to yr=2004-current

38.limit 37 to english
