# Supplementary material for: Evaluative reports on medical malpractice policies in obstetrics: a rapid scoping review
Source: Syst Rev. 2017 Sep 6;6:181. doi: 10.1186/s13643-017-0569-5 (PMC5586050; doi:10.1186/s13643-017-0569-5)
Supplement: Supplementary file 4 — Terminology. (DOCX 20 kb) [file 13643_2017_569_MOESM4_ESM.docx]

**Additional File 4. Terminology**

| **Terminology** | **Definition** | **Source** |
| --- | --- | --- |
| **Medical error^a^** | Preventable adverse effect of care. The failure of a planned action to be completed as intended or the use of a wrong plan to achieve an aim. | Report of the Quality Interagency Coordination Task Force (QuIC) to the President. *Doing What Counts for Patient Safety: Federal Actions to Reduce Medical Errors and Their Impact,* 2000 |
| **Adverse event^a^** | Undesirable and unintentional, though not necessarily unexpected, results of medical treatment. | Gale Encyclopedia of Surgery: A Guide for Patients and Caregivers  COPYRIGHT 2004 The Gale Group Inc. |
| **Damage^a^** | An injury or harm impairing the function or condition of a person. | Collins English Dictionary – Complete and Unabridged, 12th Edition 2014. (1991, 1994, 1998, 2000, 2003, 2006, 2007, 2009, 2011, 2014). Retrieved June 7 2017 from <http://www.thefreedictionary.com/damage>) |
| **Litigation costs^a^** | Includes hiring expert witnesses; the costs of a study, report, analysis, or other project ordered by the court; attorney hourly fees; court fees; copy fees; deposition fees; computer legal research services; secretarial and paralegal fees; external, consultant, and specialist fees; private investigation fees; costs in obtaining medical, school, and government records; accident reconstruction fees, and so on. | Retrieved June 7th 2017 from <http://www.legalmatch.com/law-library/article/litigation-costs-getting-the-other-side-to-pay.html#sthash.s7JLN2jv.dpuf> |
| **Cost containment^a^** | Management, control, and restriction of excessive spending. | Mosby's Medical Dictionary, 8th edition. (2009). Retrieved June 7 2017 from http://medical-dictionary.thefreedictionary.com/cost+containment |
| **Tort^a^** | An act or omission that gives rise to injury or harm to another and amounts to a civil wrong for which courts impose liability. In the context of torts, "injury" describes the invasion of any legal right, whereas "harm" describes  a loss or detriment in fact that an individual suffers. | Retrieved July 6^th^ 2017 from <https://www.law.cornell.edu/wex/tort> |

^a^ We have followed these definitions throughout this manuscript, However, we did not alter the terminology that was used in the included reports..
