# Supplementary material for: Evaluative reports on medical malpractice policies in obstetrics: a rapid scoping review
Source: Syst Rev. 2017 Sep 6;6:181. doi: 10.1186/s13643-017-0569-5 (PMC5586050; doi:10.1186/s13643-017-0569-5)
Supplement: Supplementary file 5 — Costs–descriptions by authors of included reports. (DOCX 34 kb) [file 13643_2017_569_MOESM5_ESM.docx]

# Additional File 5. Costs - Descriptions by authors of included studies

| **Author, Year** | **Description/Definition** |
| --- | --- |
| Behrens, 2011[1] | NR |
| Bovbjerg, 2005[2] | NR |
| Currie, 2008[3] | NR |
| Edwards, 2010[4] | NR |
| Ho, 2011[5] | NR |
| Iizuka, 2013[6] | NR |
| Kachalia, 2010[7] | “We defined **total liability costs** as the sum of all patient compensation and legal costs incurred by UMHS. **Patient compensation costs** included amounts paid to the patients (or families) and lien holders. **Total legal costs** were primarily defense attorney and expert fees but also included lawsuit-associated items, such as filing fees.” |
| Kilgore, 2006[8] | NR |
| Milne, 2013[9] | “**Incurred costs** included payments for the following: theHIROC lawyer; the adjuster; expert opinion; settlements paid to claimants; claimant legal costs; and, the reserve in their Ultimate Probable Cost (likely to happen).” |
| Pegalis, 2012[10] | NR |
| Santos, 2015[11] | “**Medical malpractice expenditures** are mainly due to the occurrence of preventable harm with some of the highest liability rates in obstetrics.” |
| Studdert, 2004[12] | NR |
| Thorpe, 2004[13] | NR |
| Winn, 2007[14] | NR |

**Abbreviations:** NR – not reported

**References**

1. Behrens MA. Medical liability reform: a case study of Mississippi. Obstet Gynecol. 2011;118(2 Pt 1):335-9.

2. Bovbjerg RR. Malpractice crisis and reform. Clin Perinatol. 2005;32(1):203-33.

3. Currie J, MacLeod WB. First Do No Harm? Tort Reform and Birth Outcomes. The Quarterly Journal of Economics. 2008;123(2):795-830.

4. Edwards CT. The Impact of a No-Fault Tort Reform on Physician decision-making: a look at Virgina’s Birth Injury Program. Rev Jurid Univ P R. 2010;80.

5. Ho B, Liu E. What's an Apology Worth? Decomposing the Effect of Apologies on Medical Malpractice Payments Using State Apology Laws. J Empir Leg Stud. 2011;8(S1):177-99.

6. Iizuka T. Does higher malpractice pressure deter medical errors? Journal of Law and Economics. 2013;56(1):161-88.

7. Kachalia A, Kaufman SR, Boothman R, Anderson S, Welch K, Saint S, et al. Liability claims and costs before and after implementation of a medical error disclosure program. Ann Intern Med. 2010;153(4):213-21.

8. Kilgore ML, Morrisey MA, Nelson LJ. Tort law and medical malpractice insurance premiums. Inquiry. 2006;43(3):255-70.

9. Milne JK, Walker DE, Vlahaki D. Reflections on the Canadian MORE(OB) obstetrical risk management programme. Best Pract Res Clin Obstet Gynaecol. 2013;27(4):563-9.

10. Pegalis SE, Bal BS. Closed medical negligence claims can drive patient safety and reduce litigation. Clin Orthop Relat Res. 2012;470(5):1398-404.

11. Santos P, Ritter GA, Hefele JL, Hendrich A, McCoy CK. Decreasing intrapartum malpractice: Targeting the most injurious neonatal adverse events. J Healthc Risk Manag. 2015;34(4):20-7.

12. Studdert DM, Mello MM, Brennan TA. Medical malpractice. N Engl J Med. 2004;350(3):283-92.

13. Thorpe KE. The medical malpractice 'crisis': recent trends and the impact of state tort reforms. Health Aff (Millwood). 2004;Suppl Web Exclusives:W4-20-30.

14. Winn SH. Assessing and credentialing standards of care: the UK Clinical Negligence Scheme for Trusts (CNST, Maternity). Best Pract Res Clin Obstet Gynaecol. 2007;21(4):537-55.
