# Supplementary material for: Evaluative reports on medical malpractice policies in obstetrics: a rapid scoping review
Source: Syst Rev. 2017 Sep 6;6:181. doi: 10.1186/s13643-017-0569-5 (PMC5586050; doi:10.1186/s13643-017-0569-5)
Supplement: Supplementary file 6 — Description of the strategies to mitigate litigation. (DOCX 42 kb) [file 13643_2017_569_MOESM6_ESM.docx]

**Additional File 6. Description of the strategies to mitigate litigation**

| **Author, Year (Setting)** | **Sample** | **Strategy description** | **Follow-up period** | **Adverse events** |
| --- | --- | --- | --- | --- |
| Behrens, 2011[1]  (Mississippi, USA) | 4,878 lawsuits | Name: Mississippi tort reform legislation  Description: The core of House Bill was a $500,000 limit on noneconomic damages, such as pain and suffering, applicable to most medical negligence cases. It also generally requires medical malpractice plaintiffs’ attorneys to consult with an expert before filing suit, although “a complaint, otherwise properly filed, may not be dismissed, and need not be amended, simply because the plaintiff failed to attach a certificate or waiver.” In addition, plaintiffs are required to give defendants 60 days’ written notice before commencing a medical liability lawsuit, abolished joint liability for noneconomic damages for any defendant found to be less than 30% at fault, and provides heightened pleading requirements for cases involving medical professionals who prescribe prescription drugs. | 7 years | Obstetrical/gynecological |
| Bovbjerg, 2005[2]  (USA) | 226 claims + 281 surveys | Name: Administrative compensation model  Description: A non-tort model addressing medical injuries that pays claimants for defined, medically caused harms; it is not based on fault. Such model is meant to simplify determinations of responsibility for paying compensation, limiting damage allowances, and reducing the reliance on adversary judicial process | 8 years in Florida, 9 years in Virginia | Birth-related neurologic injuries |
| Currie, 2008[3]  (USA) | NR | Name: Non-economic damage caps  Description: Noneconomic damages cover items other than monetary losses, such as pain and suffering. | NR | obstetrics |
|  |  | Name: Limit joint and several liability  Description: Under joint and several liabilities the plaintiff can recover from any individual who shares in the blame for the accident. JSL reform an indirect reform because it does not directly constrain the level of damages (Kessler and McClellan, 1996). Rather, it affects who is liable for damages. It is believed that this reform can reduce liability by removing the incentive for plaintiffs to attempt to recover from the deep pocket when there is a medical error. In practice, JSL means that if the physician makes a mistake during a delivery and the attending nurse has some culpability, then the patient may sue the nurse's employer, usually the hospital, for full damages. This is because unlike the physician, the nurse is an employee of the hospital, which is the deep pocket in the case. |  |  |
|  |  | Name: Caps on punitive damages  Description: Punitive damages are awarded in addition to compensatory (economic and noneconomic) damages in order to punish defendants for willful and wanton conduct. |  |  |
|  |  | Name: Reforms of the collateral source rule  Description: Under the common law collateral-source rule (CSR), amounts that a plaintiff receives from sources other than the defendant (e.g., from his or her own insurance) may not be admitted as evidence in a trial. |  |  |
| Edwards, 2010[4]  (Virginia, USA) | 571 obstetricians | Name: Virginia Birth-Related Neurological Injury Compensation Program (BIP)  Description: A voluntary, no-fault insurance pool. It legally precludes lawsuits for certain neurological injuries against physicians that choose to pay a yearly fee. Instead, patients must seek compensation from the BIP pool, a process that imposes little burden on physicians. In contrast, physicians that do not pay the yearly fee can be sued for these neurological injuries. The program framers never sought to overhaul the tort system generally, but instead wished to coax insurance companies to cover additional obstetricians. Consequently, they removed the cases that cause the greatest uncertainty in malpractice awards in obstetrics: birth-related neurological injuries. | 19 years | Obstetrics |
| Ho, 2011[5]  (USA) | 234,826 adverse events | Name: Apology laws  Description: These laws state that apologies or similar expressions of regret made by medical practitioners cannot be used as evidence in medical malpractice litigation. The laws are intended to protect statements of apology made by physicians in order to increase the likelihood of their use. | NR | Any medical malpractice including obstetrics |
| Iizuka, 2013[6]  (USA) | NR | Name: Caps on non-economic damages (CapsNED)  Description: Reforms concerning non-economic damages place a cap on the damages that can be awarded for non-economic losses, such as pain and suffering and emotional distress | NR | Birth trauma injury to neonate; obstetric trauma to mother |
|  |  | Name: Caps on punitive damages (CapsPD)  Description: Punitive damages are awarded to punish a defendant for intentional or malicious misconduct. Although these damages are infrequently awarded, they can be very large when granted. Punitive damage reform places a cap on these damages. |  |  |
|  |  | Name: Collateral source rule (CSR) reform  Description: The collateral source rule does not allow jury members to take into account any payments to a plaintiff other than those made by the defendant, which means that a plaintiff can recover full damages from a defendant even after the plaintiff has been compensated from other sources, including the plaintiff’s insurance or workers compensation. |  |  |
|  |  | Name: Joint and several liability reform  Description: Joint and several liability requires each liable party to be individually responsible for the entire obligation, regardless of his respective percentage of fault. Joint and several liability allows a plaintiff to seek damages from all, some, or only one of the parties alleged to have caused the injury. In many cases, a defendant can seek indemnification or reimbursement from unnamed parties. Joint and several liabilities allows plaintiffs the luxury of only needing to establish that one defendant is responsible for the injury, thereby obtaining a judgment against all defendants. The reform overturns this traditional rule to make doctors accountable for their own errors. |  |  |
| Kachalia, 2010[7]  (Michigan, USA) | 1,131 claims | Name: Medical Error Disclosure Program  Description: The program identifies patient injuries through various means, including reporting by employees, patients or family members, or patients' attorneys. It uses experienced risk managers with clinical backgrounds to lead investigations and mediate patient concerns as facts are collected, care quality is evaluated, and conclusions are disclosed. Settlements, if made, generally occur in the institution's name, in line with common practice at many institutions with closed medical staffs. | 4 years | any medical error |
| Kilgore, 2006[8]  (USA) | NR | Name: Damage caps strategy  Description: Plaintiffs in malpractice cases will typically seek to recover economic damages such as medical costs and the opportunity cost of lost income. | NR | internal medicine, surgery, obstetrics |
|  |  | Name: Statute of Limitations  Description: This strategy specifies a limited time period from occurrence or discovery of an allegedly torturous act to the filing of a claim in court. |  |  |
| Milne, 2013[9]  (Canada) | 39 participating hospitals | Name: Managing Obstetrical Risk Efficiently (MORE)  Description: Consists of three educational modules, each about 12 months in length: ‘Learning together’, “Working together’ and ‘Changing culture’. The modules teach core obstetric content to ensure all members of an obstetric unit (e.g., nurses, midwives, family physicians and obstetricians) have a similar foundation of clinical knowledge. Learning activities focus on establishing characteristics of high-reliability organizations within obstetric units, such as, safety being the first priority and everyone’s responsibility, teamwork, communication and risk-management proficiency. The program is delivered partly on site and partly on-line. A hospital-selected multidisciplinary healthcare practitioner team is first trained by Salus MORE facilitators and then supported by Salus program consultants. The end goal for the program was to change the culture of blame to a culture of patient safety. | 8 years (costs) & 9 years (catastrophic infant claims) | obstetrics |
| Pegalis, 2012[10]  (USA) | NA | Name: Patient safety guidelines  Description: Guidelines developed by examining medical malpractice claims data to identify errors that proved amenable to patient safety guidelines and protocols that ultimately helped drive down the costs and incidence of medical malpractice litigation. | NR | obstetrics |
| Santos, 2015[11]  (USA) | 5 labor and delivery sites | Name: Risk reduction labor and delivery model  Description: A new multilevel integrated practice and coordinated communication model that consisted of 4 key components: instituting new practice bundles for non-reassuring fetal status and shoulder dystocia occurrences with training for physicians and nurses; standardizing and requiring documentation of these bundles; establishing an unintended event disclosure policy, process, and training; and providing rapid feedback to teams on the model’s performance measures. These components were developed using the High Reliability Organization framework, which is a set of concepts that hospitals use to “radically reduce system failures and effectively respond when failures do occur.” High Reliability Organization framework, is a set of concepts that hospitals use to “radically reduce system failures and effectively respond when failures do occur". | 2 years and 3 months | obstetrics: labour and delivery events (shoulder dystocia and fetal distress) |
| Studdert, 2004[12]  (California, USA) | 152 cases | Name: California’s Medical Injury Compensation Reform Act (MICRA) cap  Description: The cap established under MICRA pertains to noneconomic damages only. Any action for injury against a health care provider based on “professional negligence” is subject to the cap, regardless of the severity of injury, the plaintiff’s age, or the magnitude of economic loss. Intentional torts, such as battery and sexual misconduct, are exempt. Several other features of the MICRA cap are noteworthy. First, the cap was fixed at $250,000 in 1975; unlike damages caps in some other states, it has never been adjusted for inflation. If adjusted for inflation, the cap would have reached $877,000 in 2002. Second, the cap is applied after juries have decided on damages. Therefore, juries are theoretically “blinded” to the existence of the cap in reaching their verdict. Third, any modifications to awards based on comparative fault are made before the cap is applied. Fourth, in cases that involve ongoing or future losses, a present value is assigned to the compensation. | 17 years | surgical errors, obstetrical errors, missed or delayed diagnoses, and drug errors |
| Thorpe, 2004[13]  (USA) | NR | Name: Award cap strategy  Description: Damages in medical malpractice cases fall into three general categories: noneconomic damages (pain, suffering, anguish), economic damages (lost wages and medical care expenses), and punitive damages, if conduct is viewed as malicious or in reckless disregard of plaintiffs’ rights (these are rarely awarded). | NR | obstetrics, surgical, internal |
| Winn, 2007[14]  (UK) | 7,352 obstetrics & gynaecology claims | Name: Clinical Negligence Scheme for Trusts  Description: Clinical Negligence Scheme for Trusts provides NHS trusts with a set of risk management standards for maternity services. These standards are designed to act as a framework, bringing focus to the development and implementation of clinical governance, thereby improving patient care. The standards were grounded in areas of practice that were known to give rise to litigation. The standards cover a range of both reactive and proactive risk management systems and processes, and each standard is set at three levels. Level 1 requires the establishment of a basic risk management framework and the functioning of some systems at a basic level. Level 2 requires implementation of risk management systems and processes and integration into practice. Level 3 is more demanding and requires a high level of compliance with activities such as training, the audit of systems, and evidence of changes and improvements made as a result. The areas and services included in the assessment are antenatal, intra-partum and postnatal services, midwifery-led care, obstetric anesthetics and obstetric ultrasonography. | 3 years | any medical malpractice obstetrics |

**Abbreviations:** NA – Not applicable; NR – Not reported

**References**

1. Behrens MA. Medical liability reform: a case study of Mississippi. Obstet Gynecol. 2011;118(2 Pt 1):335-9.

2. Bovbjerg RR. Malpractice crisis and reform. Clin Perinatol. 2005;32(1):203-33.

3. Currie J, MacLeod WB. First Do No Harm? Tort Reform and Birth Outcomes. The Quarterly Journal of Economics. 2008;123(2):795-830.

4. Edwards CT. The Impact of a No-Fault Tort Reform on Physician decision-making: a look at Virgina’s Birth Injury Program. Rev Jurid Univ P R. 2010;80.

5. Ho B, Liu E. What's an Apology Worth? Decomposing the Effect of Apologies on Medical Malpractice Payments Using State Apology Laws. J Empir Leg Stud. 2011;8(S1):177-99.

6. Iizuka T. Does higher malpractice pressure deter medical errors? Journal of Law and Economics. 2013;56(1):161-88.

7. Kachalia A, Kaufman SR, Boothman R, Anderson S, Welch K, Saint S, et al. Liability claims and costs before and after implementation of a medical error disclosure program. Ann Intern Med. 2010;153(4):213-21.

8. Kilgore ML, Morrisey MA, Nelson LJ. Tort law and medical malpractice insurance premiums. Inquiry. 2006;43(3):255-70.

9. Milne JK, Walker DE, Vlahaki D. Reflections on the Canadian MORE(OB) obstetrical risk management programme. Best Pract Res Clin Obstet Gynaecol. 2013;27(4):563-9.

10. Pegalis SE, Bal BS. Closed medical negligence claims can drive patient safety and reduce litigation. Clin Orthop Relat Res. 2012;470(5):1398-404.

11. Santos P, Ritter GA, Hefele JL, Hendrich A, McCoy CK. Decreasing intrapartum malpractice: Targeting the most injurious neonatal adverse events. J Healthc Risk Manag. 2015;34(4):20-7.

12. Studdert DM, Mello MM, Brennan TA. Medical malpractice. N Engl J Med. 2004;350(3):283-92.

13. Thorpe KE. The medical malpractice 'crisis': recent trends and the impact of state tort reforms. Health Aff (Millwood). 2004;Suppl Web Exclusives:W4-20-30.

14. Winn SH. Assessing and credentialing standards of care: the UK Clinical Negligence Scheme for Trusts (CNST, Maternity). Best Pract Res Clin Obstet Gynaecol. 2007;21(4):537-55.
