# Supplementary material for: Evaluative reports on medical malpractice policies in obstetrics: a rapid scoping review
Source: Syst Rev. 2017 Sep 6;6:181. doi: 10.1186/s13643-017-0569-5 (PMC5586050; doi:10.1186/s13643-017-0569-5)
Supplement: Supplementary file 7 — Initiatives for improving mdical malpractice definitions. (DOCX 17 kb) [file 13643_2017_569_MOESM7_ESM.docx]

# Additional File 7. Initiatives for improving medical malpractice definitions

| **Initiatives** | **Definitions** |
| --- | --- |
| No-fault approach | Strategies when medical injuries are compensated without proof of fault |
| Patient safety initiatives | Frameworks and system models aimed to reduce medical malpractice and ultimately clinical negligence claims at the population level |
| Communication and resolution | Strategies that involve communication between physician and patient outside the court setting to reach a mutual agreement to resolve the dispute and fair compensation |
| Caps on compensation and attorney fees | Strategies that limit the amount of non-economic or punitive damages that may be awarded for a case |
| Alternative payment system and liabilities | Strategies that reduce the burden of liability pressure and financial burden of claims payment |
| Limitations on litigation | Strategies that limit the type and amount of medical malpractice claims entering the system |
| Multi-component models | Combination of the aforementioned strategies |
