# Supplementary material for: Evaluative reports on medical malpractice policies in obstetrics: a rapid scoping review
Source: Syst Rev. 2017 Sep 6;6:181. doi: 10.1186/s13643-017-0569-5 (PMC5586050; doi:10.1186/s13643-017-0569-5)
Supplement: Supplementary file 8 — Evaluation of strategies to reduce litigation. (DOCX 45 kb) [file 13643_2017_569_MOESM8_ESM.docx]

**Additional File 8. Evaluation of strategies to reduce litigation**

| **Author, Year** | **Setting** | **Name of the strategy or program** | **Data collection methods** | **Evaluation measures** |
| --- | --- | --- | --- | --- |
| **No Fault Approach** | | | | |
| Edwards, 2010[[1](#_ENREF_1)] | Virginia, USA | Virginia Birth-Related Neurological Injury Compensation Program (BIP) | Physician-specific adjusted caesarean rate, physician participation in BIP | Virginia Health Information (V.H.I.), a non-profit public/private partnership, The dataset uses 2006 obstetrical delivery statistics that hospitals are legally required to report to V.H.I. |
| Bovbjerg, 2005[[2](#_ENREF_2)] | USA | Administrative compensation model | Closed malpractice claims and survey | administrative data |
| **Patient Safety Initiatives** | | | | |
| Milne, 2013[[3](#_ENREF_3)] | Canada | Managing Obstetrical Risk Efficiently (MORE) | Clinical core content knowledge; behavioural change and liability claims | Survey |
| Pegalis, 2012[[4](#_ENREF_4)] | USA | Patient safety guidelines | Safety | Closed claims |
| Santos, 2015[[5](#_ENREF_5)] | USA | Risk reduction labor and delivery model | Rate of event reporting and high-risk malpractice event rate per 1000 births | Medical liability risk and administrative data sets were analyzed, interviews |
| Winn, 2007[[6](#_ENREF_6)] | UK | Clinical Negligence Scheme for Trusts | Contribution calculation for maternity includes the number of births, and a trust will earn discounts as a part of the contribution assessment of maternity services, number and cost of claims | Hospital administrative records, claims data |
| **Communication and Resolution** | | | | |
| Ho, 2011[[7](#_ENREF_7)] | USA | Apology laws | Claim severity | Data are drawn from the npdb database |
| Kachalia, 2010[[8](#_ENREF_8)] | Michigen, USA | Medical Error Disclosure Program | Number of new claims for compensation, number of claims compensated, time to claim resolution, and claims-related costs. | Two data sets were linked: the UMHS risk management database (which contains claims-related performance data, such as injury and disposition dates, disposition status, and liability costs) and the Clinical Information & Decision Support Services database. |
| **Caps of compensation and attorney fees** | | | | |
| Behrens, 2011[[9](#_ENREF_9)] | Mississippi, USA | Mississippi tort reform legislation | Data regarding lawsuits against physicians insured by the Medical Assurance Company of Mississippi (MACM) | Medical Assurance Company of Mississippi |
| Iizuka, 2013[[10](#_ENREF_10)] | USA | Caps on non-economic damages (CapsNED) | Patient safety indicators - estimation of three models to examine the relationship between tort reforms and preventable medical complications. | Nationwide Inpatient Sample (NIS), information on state tort reform (the second data set) |
|  |  | Caps on punitive damages (CapsPD) |  |  |
|  |  | Collateral source rule (CSR) reform |  |  |
| Currie, 2008[[11](#_ENREF_11)] | USA | Non-economic damage caps | C-section; nonpreventable complications (e.g., breech delivery, cephalopelvic disproportion); preventable complications (e.g., fever, meconium); 5-minute APGAR <8; low birth weight | Vital Statistics natality data. These data come from birth certificates collected by each state and filed with the National Center for Health Statistics |
| Kilgore, 2006[[12](#_ENREF_12)] | USA | Damage caps | Insurer investment returns on physician malpractice insurance premiums | Medical Liability Monitor (MLM 2005) annual survey of physician insurers. MLM identifies the largest malpractice insurance providers in each state and repeatedly contacts them to provide premium data. Except in states with a single provider, MLM reports premiums for between two and seven firms. |
| Studdert, 2004[[13](#_ENREF_13)] | California, USA | California’s Medical Injury Compensation Reform Act (MICRA) cap | Absolute reductions in noneconomic damages under the cap; proportional reductions in noneconomic damages under the cap | California Jury Verdicts Weekly (CJVW) - a publication that covers cases statewide. The reports are obtained through a combination of voluntary reporting by and CJVW solicitations to attorneys, with the latter based on information gathered by CJVW staff from court dockets and from news and wire reports. |
| Thorpe, 2004[[14](#_ENREF_14)] | USA | Award cap | Trends in premiums earned and loss ratios | National Association of Insurance Commissioners (NAIC), examined trends in premiums earned and loss ratios, by state, for 1985–2001. |
| **Alternative Payment System and Liabilities** | | | | |
| Currie, 2008[[11](#_ENREF_11)] | USA | Limit Joint and Several Liability | C-section; complications (e.g., breech delivery, cephalopelvic disproportion); preventable complications; 5-minute APGAR <8; low birth weight | Vital Statistics natality data. These data come from birth certificates collected by each state and filed with the National Center for Health Statistics. |
| Iizuka, 2013[[10](#_ENREF_10)] | USA | joint and several liability (JSL) reform | Patient safety indicators - estimation of three models to examine the relationship between tort reforms and preventable medical complications | Nationwide Inpatient Sample (NIS), information on state tort reform (the second data set) |
| **Limitations on litigation** | | | | |
| Kilgore, 2006[[12](#_ENREF_12)] | USA | Statute of Limitations | Insurer investment returns on physician malpractice insurance premiums | Medical Liability Monitor (MLM 2005) annual survey of physician insurers. Every year, MLM identifies the largest malpractice insurance providers in each state and repeatedly contacts them to provide premium data. Except in states with a single provider, MLM reports premiums for between two and seven firms. Data on the market shares of each firms are not made available per agreement with the firms. These  data include 1991–2004 state- and substate level premiums for three specialties: obstetrics/ gynecology, general surgery, and internal medicine. |

**References**

1. Edwards CT. The Impact of a No-Fault Tort Reform on Physician decision-making: a look at Virgina’s Birth Injury Program. Rev Jurid Univ P R. 2010;80.

2. Bovbjerg RR. Malpractice crisis and reform. Clin Perinatol. 2005;32(1):203-33.

3. Milne JK, Walker DE, Vlahaki D. Reflections on the Canadian MORE(OB) obstetrical risk management programme. Best Pract Res Clin Obstet Gynaecol. 2013;27(4):563-9.

4. Pegalis SE, Bal BS. Closed medical negligence claims can drive patient safety and reduce litigation. Clin Orthop Relat Res. 2012;470(5):1398-404.

5. Santos P, Ritter GA, Hefele JL, Hendrich A, McCoy CK. Decreasing intrapartum malpractice: Targeting the most injurious neonatal adverse events. J Healthc Risk Manag. 2015;34(4):20-7.

6. Winn SH. Assessing and credentialing standards of care: the UK Clinical Negligence Scheme for Trusts (CNST, Maternity). Best Pract Res Clin Obstet Gynaecol. 2007;21(4):537-55.

7. Ho B, Liu E. What's an Apology Worth? Decomposing the Effect of Apologies on Medical Malpractice Payments Using State Apology Laws. J Empir Leg Stud. 2011;8(S1):177-99.

8. Kachalia A, Kaufman SR, Boothman R, Anderson S, Welch K, Saint S, et al. Liability claims and costs before and after implementation of a medical error disclosure program. Ann Intern Med. 2010;153(4):213-21.

9. Behrens MA. Medical liability reform: a case study of Mississippi. Obstet Gynecol. 2011;118(2 Pt 1):335-9.

10. Iizuka T. Does higher malpractice pressure deter medical errors? Journal of Law and Economics. 2013;56(1):161-88.

11. Currie J, MacLeod WB. First Do No Harm? Tort Reform and Birth Outcomes. The Quarterly Journal of Economics. 2008;123(2):795-830.

12. Kilgore ML, Morrisey MA, Nelson LJ. Tort law and medical malpractice insurance premiums. Inquiry. 2006;43(3):255-70.

13. Studdert DM, Mello MM, Brennan TA. Medical malpractice. N Engl J Med. 2004;350(3):283-92.

14. Thorpe KE. The medical malpractice 'crisis': recent trends and the impact of state tort reforms. Health Aff (Millwood). 2004;Suppl Web Exclusives:W4-20-30.
