# Supplementary material for: Evaluative reports on medical malpractice policies in obstetrics: a rapid scoping review
Source: Syst Rev. 2017 Sep 6;6:181. doi: 10.1186/s13643-017-0569-5 (PMC5586050; doi:10.1186/s13643-017-0569-5)
Supplement: Supplementary file 9 — Outcomes of the strategies to reduce litigation. (DOCX 48 kb) [file 13643_2017_569_MOESM9_ESM.docx]

**Additional File 9. Outcomes of the strategies to reduce litigation**

| **Author, Year** | **Setting** | **Name of the strategy or program** | **Clinical outcomes** | **Advantages** | **Cost Savings** |
| --- | --- | --- | --- | --- | --- |
| **No Fault Approach** | | | | |  |
| Edward, 2010[[1](#_ENREF_1)] | Virginia, USA | Virginia Birth-Related Neurological Injury Compensation Program (BIP) | Virginia’s tort reform shields participating physicians almost entirely from the negative effects of malpractice claims for certain injuries; results do not support the theory of reduced defensive medicine and provide at most mild evidence suggesting that the Birth Injury Program induces physicians to practice less defensively | BIP participating physicians are shielded against malpractice liability for covered injuries as patients eligible for BIP are prohibited from filing any lawsuits related to their injuries; BIP coverage also provides physicians with substantial time savings (i.e., program informs physicians of claims filed against them; however, they play no role in the proceedings other than turning over medical records and are not informed of the results) | Obstetricians in Virginia enjoyed relatively low malpractice premiums when compared to national rates |
| Bovbjer, 2005[[2](#_ENREF_2)] | USA | Administrative compensation model | Administrative data of closed malpractice claims and survey revealed that the programs, as intended, kept obstetric liability coverage available and decreased tort premiums. Administrative claims were much lower than expected (196 during the first 8 years in Florida, 30 in 9 years in Virginia), not unaffordably numerous as some opponents of reform had claimed. | Decreased tort premiums; administrative claims were much lower; claims resolution was fast and once claims were filed (a median time to resolution of 148 days from filing, compared with 591 days in tort), but the time from injury to filing was the same in both systems. | Administrative costs (overhead) were low (10.3 percent vs. 46.9 percent) |
| **Patient Safety Initiatives** | | | | |  |
| Milne, 2013[[3](#_ENREF_3)] | Canada | Managing Obstetrical Risk Efficiently (MORE) | Patient safety showed the highest average increase over an incremental time period, with a 20 percent increase; liability claims: a significant reduction (P < 0.001) was shown in average incurred costs in the obstetrics labour and delivery units after the onset of the program | A robust, flexible goal-setting process for use in all hospital settings in all geographic environments, as well as a strong evaluation process to measure the program’s impact and be able to demonstrate the return on investment for each client hospital. | A significant reduction(P<0.001) was shown in average incurred costs in the obstetrics labour and delivery units after the onset of the program |
| Pegalis, 2012[[4](#_ENREF_4)] | USA | Patient safety guidelines | Incidence of anesthesia-related deaths dropped from one to two per 10,000 anesthetic procedures to one for every 200,000 procedures. Also, improved perinatal outcomes were reported with lower maternity and fetal injury rates, primary cesarean delivery rates, and litigation rates | Improves safety, lessens litigation, decreased deaths, reduction in medical malpractice insurance premiums, happier profession | The average compensation payment decreased from more than $27 million to approximately $2.5 million per year and serious adverse events such as death, permanent or severe temporary harm was reduced from five events per year to zero events. |
| Santos, 2015[[5](#_ENREF_5)] | USA | Risk reduction labor and delivery model | Although any defensive practices were encouraged during the study period, it was noted that the caesarean delivery rate increased over time, in step with national trends. Also, 50 percent reduction in shoulder dystocia and fetal distress cases | Reporting of unintended events increased significantly, high-risk malpractice events decreased significantly | NR |
| Winn, 2007[[6](#_ENREF_6)] | UK | Clinical Negligence Scheme for Trusts | There are no definitive data to demonstrate that the CNST Maternity Standards have made a difference to patients when measured by outcomes such as claims | Decrease in the number of maternity claims as a percentage of total clinical negligence | Decrease in cost of maternity claims as a percentage of total clinical negligence claims |
| **Communication and Resolution** | | | | |  |
| Ho, 2011[[7](#_ENREF_7)] | USA | Apology laws | The effect of apology laws on the size of payment: apologies are most valuable for cases involving obstetrics and anesthesia, for cases involving infants, and for cases involving improper management by the physician and failures to diagnose. | Apologies have substantial value either as evidence for the courts, or as a mechanism that helps alleviate a patient’s demands for restitution | The passage of the apology law accounts for a $32,342 (12.8 percent) decrease in the size of malpractice payments |
| Kachalia, 2010[[8](#_ENREF_8)] | Michigen, USA | Medical Error Disclosure Program | Average monthly rate of new claims decreased from 7.03 to 4.52 per 100 000 patient encounters (rate ratio [RR], 0.64 [95% CI, 0.44 to 0.95])  Average monthly rate of lawsuits decreased from 2.13 to 0.75 per 100 000 patient encounters (RR, 0.35 [CI, 0.22 to 0.58])  Median time from claim reporting to resolution decreased from 1.36 to 0.95 years  Average monthly cost rates decreased for total liability (RR, 0.41 [CI, 0.26 to 0.66]), patient compensation (RR, 0.41 [CI, 0.26 to0.67]), and non–compensation-related legal costs (RR, 0.39 [CI, 0.22 to 0.67]) | Showed that medical center can implement a disclosure-with-offer program without increasing malpractice costs; a disclosure program may address some of the main shortcomings of our current liability system, namely shortening long waits for compensation and decreasing administrative expenses; disclosure may actually reduce another inefficiency of the malpractice system: preventing both meritorious and nonmeritorious claims from becoming expensive lawsuits; the lower number of paid claims after implementation may suggest that disclosure with offer may not always ensure that injured patients receive compensation. This finding, however, may challenge past assumptions that everyone who had a harmful error expects compensation. The UMHS experience demonstrates that disclosure with offer can be conducted—in a setting similar to many other centers in the United States—without exacerbating liability costs. | Average monthly rate of new claims decreased from 7.03 to 4.52 per 100 000 patient encounters (rate ratio [RR], 0.64 [95% CI, 0.44 to 0.95]);  average monthly rate of lawsuits decreased from 2.13 to 0.75 per 100 000 patient encounters (RR, 0.35 [CI, 0.22 to 0.58]);  median time from claim reporting to resolution decreased from 1.36 to 0.95 years;  average monthly cost rates decreased for total liability (RR, 0.41 [CI, 0.26 to 0.66]); patient compensation (RR, 0.41 [CI, 0.26 to0.67]), and non–compensation-related legal costs (RR, 0.39 [CI, 0.22 to 0.67]) |
| **Caps of compensation and attorney fees** | | | | |  |
| Behrens, 2011[[9](#_ENREF_9)] | Mississippi, USA | Mississippi tort reform legislation | The number of MACM-insured Physicians increased in Mississippi after the implementation of tort reform.; although tort reforms (particularly the limit on noneconomic damages and pursuit notice) reportedly have enabled MACM to resolve some claims more easily, these reforms have also significantly reduced the frequency of both claims and lawsuits. | Medical liability insurance premiums for MACM-insured physicians have been both reduced and refunded each year for the past 5 years (2006–2010) | Doctors covered by MACM did not receive an increase in premiums in 2005; premiums were reduced, and refunds were given each year from 2006 to 2010 |
| Iizuka, 2013[[10](#_ENREF_10)] | USA | Caps on non-economic damages (CapsNED) | CSR reform are mixed and not necessarily consistent with the predicted impact. For example, in only one case were CapsNED associated with a higher probability of medical errors. However, these comparisons are only suggestive because many factors, including state fixed-effects, are not yet controlled | Expected to reduce the malpractice liability pressure that medical providers face, which, in turn, may increase preventable medical errors. | NR |
|  |  | Caps on punitive damages (capsPD) | States with CapsPD have more medical errors than the states without these caps | Reduces the malpractice liability pressure that medical providers face |  |
|  |  | Collateral source rule (CSR) reform | The results for CapsNED and CSR reform are mixed and not necessarily consistent with the predicted impact. For example, in only one case were CapsNED associated with a higher probability of medical errors. However, these comparisons are only suggestive | Collateral source rule reform either permits or requires courts to reduce awards by the amount paid to the plaintiff by collateral sources, which is likely to reduce the liability pressure on the medical provider |  |
| Currie, 2008[[11](#_ENREF_11)] | USA | Non-economic damage caps | Caps on noneconomic damages increase preventable complications by 6% | A model that analyzes the incentives created by specific tort reforms and explores the effect of tort reform on both the level of care and procedure use during child birth. Our model shows that contrary to popular belief, reducing the threat of malpractice can increase the use of procedures, such as C-sections, and may reduce the effort made by doctors in realistic scenarios. Second, we have assembled very detailed data on tort reform in an effort to accurately identify changes in the laws. We apply these data to a large national panel data set covering an important population, newborns and their mothers, and examine a range of outcomes representing procedure use, care taken by physicians, and maternal and infant health. Finally, we show that our results are robust to many specification checks. | Caps on damages are found to increase procedure use, and hence costs. |
| Kilgore, 2006[[12](#_ENREF_12)] | USA | Damage caps | NR | NR | Caps on noneconomic damages can significantly constrain the growth of medical malpractice premiums. The introduction of a cap lowered obstetrics/gynecology premiums by 25.5% (p<0.001). Also, an increase of $100,000 in the statutory cap on noneconomic damages increased malpractice premiums by 3.9% (p<0.001) |
| Studdert, 2004,[[13](#_ENREF_13)] | California, USA | California’s Medical Injury Compensation Reform Act (MICRA) cap | Absolute reductions in noneconomic damages under the cap. The mean reductions for grave injury were seven times larger than those for minor injury; the differences in medians for these two levels of injury differed by a factor of three.  Proportional reductions in noneconomic damages under the cap. Examining reductions imposed by the cap in terms of the percentage decrease in the plaintiffs’ original award, rather than in absolute terms, they found huge variations across verdicts. Reductions ranged from 2 percent to 82 percent (mean = 37 percent, standard deviation, ±20 percent) for nonfatal injuries and from 6 percent to 88 percent (mean = 47 percent, SD, ±24 percent) for fatal injuries. Severe neurological injuries to newborns were common in the first group, accounting for nine of the twenty verdicts with the smallest percentage reductions. Injuries that caused pain or disfigurement but not significant loss of physical functioning dominated the second group: Twelve of the twenty verdicts with the largest reductions fit this profile. In addition, seven of the twenty largest reductions involved unnecessary or repeated surgical procedures, and four involved injuries to the female breast. The balance between economic and noneconomic components of the award is critical. Noneconomic damages constitute 10 percent or less of the overall award in verdicts with proportionally small reductions, but they account for the vast majority of awards with the largest reductions. Because verdicts for injuries such as deafness, numbness, disfigurement, and chronic pain attracted relatively small economic damages awards, imposition of the cap eliminated most of the award. Female and elderly plaintiffs. In unadjusted analyses, we found no significant differences in the percentage reductions experienced by female and male litigants after application of the MICRA cap (41 percent versus 38 percent reductions, respectively; t = –0.9, p = .4). To focus this comparison on the class of plaintiffs at highest risk of exhibiting sex disparities, newborns were excluded and the elderly from comparison; there still was no significant difference. | Caps advance the horizontal equity of compensation; they completely eliminate dispersion of awards above the limit of the cap | NR |
| Thorpe, 2004[[14](#_ENREF_14)] | USA | Award cap | NR | NR | The empirical results indicate that the caps on awards adopted by several states were associated with lower loss ratios and lower premiums.  -Loss ratios in states capping awards were 11.7% lower than in states without caps  -Loss ratios were 13.3 percent lower in, states with discretionary collateral offsets  -Loss ratios were 25 percent lower in states that adopted both damge cap and collateral source reforms  -Premiums in states with a cap on awards were 17.1 percent lower than in states without such caps  All other tort reforms (punitive damage cap, mandatory collateral offset rule, attorney fee caps) found no signficant effect |
| **Alternative Payment System and Liabilities** | | | | |  |
| Currie, 2008[[11](#_ENREF_11)] | USA | Limit Joint and Several Liability | Reduces C-sections, reduces preventable complications of labor and delivery by 13% | The model shows that contrary to popular belief, reducing the threat of malpractice can increase the use of procedures, such as C-sections, and may reduce the effort made by doctors in realistic scenarios. | NR |
| Iizuka, 2013[[10](#_ENREF_10)] | USA | joint and several liability (JSL) reform | States with JSL reform have fewer medical errors than states without the reform | NR | NR |
| **Limitations on litigation** | | | | |  |
| Kilgore, 2006[[12](#_ENREF_12)] | USA | Statute of Limitations | NR | NR | Increasing the length of the statute of limitations decreases premiums and mandatory collateral source offsets increase premiums appear to be artifacts of the timing of these laws in a small handful of states. It is likely that these measures were simply ineffective rather than causing a rise in premiums. |

**References**

1. Edwards CT. The Impact of a No-Fault Tort Reform on Physician decision-making: a look at Virgina’s Birth Injury Program. Rev Jurid Univ P R. 2010;80.

2. Bovbjerg RR. Malpractice crisis and reform. Clin Perinatol. 2005;32(1):203-33.

3. Milne JK, Walker DE, Vlahaki D. Reflections on the Canadian MORE(OB) obstetrical risk management programme. Best Pract Res Clin Obstet Gynaecol. 2013;27(4):563-9.

4. Pegalis SE, Bal BS. Closed medical negligence claims can drive patient safety and reduce litigation. Clin Orthop Relat Res. 2012;470(5):1398-404.

5. Santos P, Ritter GA, Hefele JL, Hendrich A, McCoy CK. Decreasing intrapartum malpractice: Targeting the most injurious neonatal adverse events. J Healthc Risk Manag. 2015;34(4):20-7.

6. Winn SH. Assessing and credentialing standards of care: the UK Clinical Negligence Scheme for Trusts (CNST, Maternity). Best Pract Res Clin Obstet Gynaecol. 2007;21(4):537-55.

7. Ho B, Liu E. What's an Apology Worth? Decomposing the Effect of Apologies on Medical Malpractice Payments Using State Apology Laws. J Empir Leg Stud. 2011;8(S1):177-99.

8. Kachalia A, Kaufman SR, Boothman R, Anderson S, Welch K, Saint S, et al. Liability claims and costs before and after implementation of a medical error disclosure program. Ann Intern Med. 2010;153(4):213-21.

9. Behrens MA. Medical liability reform: a case study of Mississippi. Obstet Gynecol. 2011;118(2 Pt 1):335-9.

10. Iizuka T. Does higher malpractice pressure deter medical errors? Journal of Law and Economics. 2013;56(1):161-88.

11. Currie J, MacLeod WB. First Do No Harm? Tort Reform and Birth Outcomes. The Quarterly Journal of Economics. 2008;123(2):795-830.

12. Kilgore ML, Morrisey MA, Nelson LJ. Tort law and medical malpractice insurance premiums. Inquiry. 2006;43(3):255-70.

13. Studdert DM, Mello MM, Brennan TA. Medical malpractice. N Engl J Med. 2004;350(3):283-92.

14. Thorpe KE. The medical malpractice 'crisis': recent trends and the impact of state tort reforms. Health Aff (Millwood). 2004;Suppl Web Exclusives:W4-20-30.
